# Cross‐sectional study of interprofessional collaboration among Japanese nurse practitioners

**DOI:** 10.1002/jgf2.70062

**Published:** 2025-09-16

**Authors:** Naoko Tokunaga, Akihiro Araki, Hiromi Fukuda

**Affiliations:** ^1^ Graduate School of Nursing Science Oita University of Nursing and Health Sciences Oita Japan; ^2^ Social Medical Corporation Daito Yotsuba Hospital Oita Japan; ^3^ Oita University of Nursing and Health Sciences Oita Japan

**Keywords:** collaboration, competency, interprofessional, nurse practitioners, support, workplace

## Abstract

**Background:**

While interprofessional collaboration is becoming increasingly important, its current status and the competency of nurse practitioners (NPs) to collaborate with multiple professionals in Japan have not been studied. This study aimed to clarify and examine the current status of interprofessional collaboration in the workplace for Japanese NPs and its related factors.

**Methods:**

For this cross‐sectional study, 760 Japanese NPs who had passed the NP qualification exam conducted by the Japanese Organization NP Faculties were targeted. Data were collected between July and December 2023, using the Assessment of Interprofessional Team Collaboration Scale‐II‐J (AITCS‐II‐J), the Japanese version of the Self‐assessment Scale of Interprofessional Competency (JASSIC), and the Workplace Support Scale. Descriptive statistics and multiple regression analyses were used for data analysis.

**Results:**

Of the 760 targeted, 137 participated, indicating a response rate of 18.0% (100% valid response rate). The AITCS‐II‐J was associated with the JASSIC and Workplace Support Scale. It was also associated with the JASSIC subscales of “Patient‐/Client‐/Family‐/Community‐Centered” and “Facilitation Relationship.” The JASSIC was associated with “Informational Support” and “Evaluative Support.”

**Conclusions:**

These results indicated that NPs' interprofessional collaboration skills and workplace support were the main factors determining collaboration quality. Additionally, informational and evaluative support from superiors was crucial to improve NPs' ability to collaborate with other professionals. To achieve effective interprofessional collaboration, developing advanced clinical and communication skills among NPs is necessary.

## 1 INTRODUCTION

The global population is aging rapidly, and Japan specifically has one of the highest rates of aging in the world.^1^ To support older adult medical care, effective collaboration between healthcare professionals is essential.^2^ Older adults generally have multiple chronic conditions and require continuous care from different healthcare professionals. Therefore, lack of interprofessional collaboration could negatively affect older patient outcomes.^2^ As the shortage of healthcare professionals is becoming a growing issue in various countries and regions, interprofessional collaboration, in which healthcare professionals cooperate and work together effectively, is becoming increasingly important.^3^

Studies have revealed that interprofessional collaboration improves patient outcomes, reduces medical costs, and enhances the quality of medical care.^4^, ^5^ Therefore, promoting it is essential for improving older adult healthcare in medical settings with staff shortage.^6^, ^7^ To this end, studies have developed scales to evaluate the current status of interprofessional collaboration and healthcare professionals' abilities,^7^, ^8^, ^9^ the results of which help examine the promotion of multidisciplinary collaboration. Such studies have been conducted in various occupations and settings, including nursing.^10^, ^11^

As the importance of interprofessional collaboration increases, nurses are expected to take leadership roles within healthcare teams in order to provide efficient and effective care.^12^ Among them, advanced practice nurses (APNs) have interprofessional collaboration skills as a competency and provide advanced nursing care in collaboration with various professionals.^13^ APNs generally have graduate‐level education in advanced nursing practice and are represented by nurse practitioners (NPs) and clinical nurse specialists (CNSs).^13^ APNs' activities have different characteristics, and NPs work as generalists who intervene in various settings, such as hospitals and homes, targeting a diverse range of patients.^14^ On the other hand, CNSs mainly work as specialists in hospitals and healthcare institutions, targeting specific patients in their areas of expertise, which differs from NPs' role.^14^, ^15^ As generalists, NPs work with a wide range of patients, coordinating with other professionals, educating staff, reducing the workload of other professionals, integrating care, and promoting patient participation in care.^15^ In addition, they serve as a hub for interprofessional teams.^16^, ^17^ Furthermore, they play a role in providing consistent medical care to patients and staff in a workplace where interprofessional team members change.^16^ In this way, NPs are expected to play a key role in team‐based medical care.

Similar to NPs in other countries, Japanese NPs also engage in generalist activities targeting various patients through interprofessional collaboration.^18^ Additionally, based on the specialized nursing practice training system, they perform advanced, specific medical tasks.^19^ These advanced specific medical tasks are unique to Japan and are performed under the direction of a physician,^19^ requiring collaboration with physicians and other professionals. In Japan, where the population is aging and birth rates are declining, there are regions facing a shortage of doctors. Providing medical care in such regions has become a particularly pressing issue within the country. Therefore, it is expected that Japanese NPs will work in a variety of settings, including hospitals, clinics, and home care stations, and collaborate with doctors and other professionals to quickly provide patients with the medical care they need.^20^, ^21^ In this way, Japanese NPs are expected to continue providing high‐quality medical care to patients in aging communities by collaborating with other professionals and utilizing existing systems.^22^ Therefore, it will become increasingly important for them to enhance their ability to collaborate with other professionals.

**FIGURE 1:**
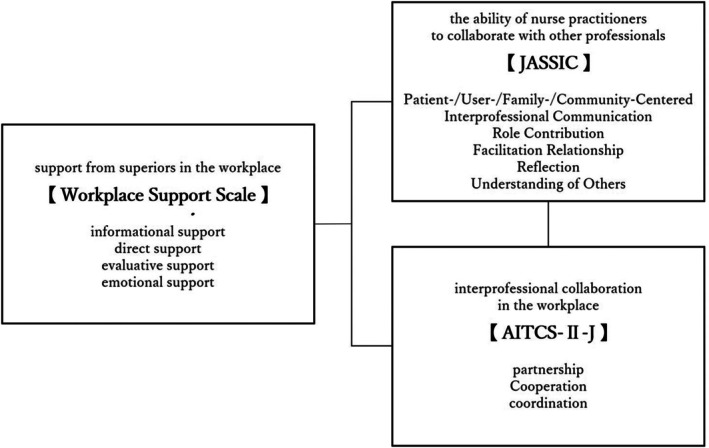
Conceptual framework.

Studies on the practice of NPs in Japan have reported the importance of workplace relationships and support systems.^17^, ^23^, ^24^ When NPs collaborate with other professionals, support from supervisors and other colleagues is crucial,^21^, ^25^ but it remains unclear what kind of support from supervisors is related to NPs' interprofessional collaboration. Furthermore, they work in hospitals, clinics, elderly care facilities, etc., and may also be assigned to the medical department with which physicians are affiliated, in addition to the nursing department.^20^, ^21^ As such, they work in diverse work environments in collaboration with various professionals.^20^, ^21^ However, no study has evaluated interprofessional collaboration in NPs' workplaces using evaluation scales. To enhance interprofessional collaboration in the workplace, it is necessary to conduct quantitative evaluations of interprofessional collaboration, clarify the current situation, and consider countermeasures.^26^ Therefore, through its conceptual framework (Figure 1), this study aims to clarify the actual state of each concept and the relationships between them, focusing on (1) NPs' workplace environments, (2) support from supervisors, (3) NPs' interprofessional collaboration skills, and (4) interprofessional collaboration in the workplace where NPs work. We hypothesized that there are correlations between each concept. Furthermore, our research questions were (1) what kind of supervisor support is related to NPs' interprofessional collaboration ability and (2) which interprofessional collaboration ability of NPs is related to interprofessional collaboration in their workplace?

## 2 METHODS

### 2.1 Study design and participants

This cross‐sectional study implemented anonymous, self‐administered questionnaires in the form of web surveys. At the time of the study, there were approximately 760 NPs in Japan,^27^ and all were targeted for the study. All the NPs had completed a master's course accredited by the Japanese Organization NP Practitioner Faculties (JONPF) and had passed the JONPF NP qualification exam to become a certified NP. Two recruitment methods were employed. The first included the researcher asking the JONPF for their cooperation and sending the request documents to the relevant study candidates via email. The second method involved using snowball sampling to send the request documents via email.

### 2.2 Data collection

The data collection period was between July 1 and December 31, 2023. Participants were asked to respond either on paper or online; if they chose paper, they were asked to respond by mail; if they chose the Internet, they were asked to complete the survey via Google Forms. To avoid duplicate responses, the survey included a confirmation question asking whether the participants had already responded.

### 2.3 Variables

#### 2.3.1 Nurse practitioners' characteristics

We surveyed NPs for several basic characteristics, including age, gender, postgraduate course, institution, department, and whether they held specialist or certified nurse qualifications. Following the studies investigating the factors related to interprofessional collaboration,^28^, ^29^ we surveyed participants' years of experience as an NP, years of experience at the institution, position, and learning experience related to interprofessional education (IPE).

#### 2.3.2 Assessment of Interprofessional Team Collaboration Scale‐II‐J

The Assessment of Interprofessional Team Collaboration Scale‐II‐J (AITCS‐II‐J), the Japanese version of the original AITCS‐II, was used as the primary scale for evaluating interprofessional collaboration in the NPs' workplaces.^9^ This version has been translated into Japanese and adapted for Japan's medical context,^8^ specifically for healthcare professionals in a variety of settings; thus, we have decided to use it.

The AITCS‐II‐J objectively and quantitatively measures the effectiveness and function of interprofessional collaboration in healthcare and comprises 23 items across 3 factors: partnership (8 items), cooperation (8 items), and coordination (7 items). Each item is rated on a 5‐point Likert scale ranging from “1: I strongly disagree” to “5: I strongly agree,” with total scores ranging from 23 to 115 points. Therefore, higher scores indicate a higher quality of interprofessional collaboration. The reliability of the Japanese version was high, with Cronbach's alpha coefficients of 0.91–0.94 for each subscale, indicating high internal consistency. Construct validity was verified in previous studies.^9^, ^30^

#### 2.3.3 Japanese version of the Self‐assessment Scale of Interprofessional Competency

The Japanese version of the Self‐assessment Scale of Interprofessional Competency (JASSIC) was used to measure the ability of NPs to collaborate with other professionals. This self‐assessment scale quantifies the abilities necessary for collaboration among healthcare professionals in Japan working in various settings; thus, we have decided to use it. It comprises 18 items across 6 factors: “Domain 1: Patient‐/User‐/Family‐/Community‐Centered” (3 items), “Domain 2: Interprofessional Communication” (3 items), “Domain 3: Role Contribution” (3 items), “Domain 4: Facilitation Relationship” (3 items), “Domain 5: Reflection” (3 items), and “Domain 6: Understanding of Others” (3 items).^7^ Each item is rated on a 5‐point Likert scale ranging from “1: I completely disagree” to “5: I completely agree,” with total scores ranging from 18 to 90 points. Higher scores indicate a higher ability for interprofessional collaboration. The scale's reliability was good, with Cronbach's alpha coefficients of 0.92 overall and 0.87–0.95 for each subscale, indicating good internal consistency. Construct validity was verified using confirmatory factor analysis (Goodness of Fit Index: GFI = 0.85, Root Mean Square Error of Approximation: RMSEA = 0.09, and the validity of the Japanese version was confirmed through a rigorous translation and cross‐cultural adaptation process).^7^

#### 2.3.4 Workplace Support Scale

The Workplace Support Scale was adopted to investigate workplace support from supervisors. This scale was developed to assess nurses in clinical settings based on the Komaki Social Support Scale; thus, we have decided to use it. It comprises 4 factors and 12 items: informational support (4 items), direct support (3 items), evaluative support (3 items), and emotional support (2 items). Each item is rated on a 5‐point Likert scale, from “1: Not at all” to “5: Always,” with total scores ranging from 12 to 60 points and higher scores indicating a higher degree of support received from one's workplace. The scale's factor validity was confirmed.^31^

### 2.4 Statistical methods

Descriptive statistics were calculated for the study variables. Additionally, to clarify the factors related to interprofessional collaboration in the NPs' workplaces, multiple regression analysis was conducted using the AITCS‐II‐J scores as the dependent variable^9^ and the JASSIC,^7^ Workplace Support Scale,^31^ years of experience as an NP, position, and learning experience related to IPE,^28^, ^29^ which was considered an important factor based on previous studies, as explanatory variables.

To examine NPs' ability to collaborate with other professionals in their workplaces, a multiple regression analysis was conducted using the AITCS‐II‐J as the dependent variable and the six subscales of the JASSIC as explanatory variables. Furthermore, to clarify workplace support related to NPs' ability to collaborate with other professionals, another multiple regression analysis was conducted, with JASSIC as the dependent variable and the four subscales of the Workplace Support Scale as explanatory variables.

In all multiple regression analyses, the variance inflation factor (VIF) values were calculated as a diagnostic for multicollinearity to verify the assumptions of regression analysis. Missing data were not treated as missing values but were used in the analysis. The results were considered statistically significant at *p* < 0.05. IBM SPSS Statistics (version 29.0 Armonk, NY, USA) was used for the analysis.

### 2.5 Ethical considerations

The study was approved by the Ethical Committee of Oita University of Nursing and Health Sciences Research (approval number 23‐54). Before participating, all participants were informed about the study purpose, content, privacy protection, data management, disposal methods, respect for free will, withdrawal of consent, and results publication. Additionally, the consent form explained in writing that the participants' responses to the questionnaire would be indicative of providing consent.

## 3 RESULTS

We received responses from 137 of approximately 760 NPs working in Japan, indicating a response rate of 18.0% (100% valid response rate).

### 3.1 Nurse practitioners' characteristics

Table 1 lists the characteristics of the NPs. The respondents' average age was 42.74 (standard deviation [SD] = 7.74). The average number of years of experience as NPs was 5.42 (SD = 3.49), and the average number of years of experience at the institution was 8.40 (SD = 8.14).

**TABLE 1:** Nurse practitioners characteristics (*N* = 137).

Variables	*n*	%
Age		
≦29	2	1.5
30–39	48	35.0
40–49	58	42.3
50–59	27	19.7
60≧	2	1.5
Gender		
Male	56	40.9
Female	81	59.1
Postgraduate course		
Critical care	75	54.7
Primary care	62	45.3
Years of experience as an NP		
≦2	40	29.4
3–4	21	15.3
5–6	20	14.6
7–8	26	19.0
9≧	30	21.9
Institution		
Hospital	114	83.2
Home healthcare (long‐term care health facility, Home Nursing Station, Clinic)	1	12.4
Other	6	4.4
Years of experience at the institution		
≦5	67	48.9
6–10	27	19.7
11–15	22	16.1
16–20	7	5.1
21≧	14	10.2
Department		
Medical department	43	31.4
Nursing department	67	48.9
Other	27	19.7
Position		
Absence	84	61.3
Presence	53	38.7
Specialist nurse qualifications		
Absence	133	97.1
Presence	4	2.9
Certified nurse qualifications		
Absence	119	86.9
Presence	18	13.1
Interprofessional professional education (IPE)		
Absence	22	16.1
Presence	115	83.9

### 3.2 Factors related to interprofessional collaboration in the workplace

Table 2 lists the factors related to interprofessional collaboration in the workplace (AITCS‐II‐J). The average total score of the AITCS‐II‐J was 71.00 (±15.59) points. The total score was related to the JASSIC (*β* = 0.31, 95% confidence interval [CI]: 0.23–0.73) and the Workplace Support Scale (*β* = 0.42, 95% CI: 0.36–0.82). The adjusted *R* ^2^ value was 0.37. As a result of verifying model assumptions, the VIF value for the explanatory variables was 1.25 for JASSIC, 1.25 for the Workplace Support Scale, 1.07 for the number of years of experience as an NP, 1.08 for the presence or absence of a position, and 1.09 for the presence or absence of IPE. Moreover, there was no evidence of multicollinearity.

**TABLE 2:** Factors related to interprofessional collaboration in the workplace (*N* = 137).

Variable	*B*	SE	*β*	95% CI	
				LL	UL
JASSIC^a^	0.48	0.12	0.31*	0.23	0.73
Workplace Support Scale^b^	0.58	0.11	0.42*	0.36	0.81
Years of experience as an NP	−0.04	0.33	−0.01	−0.69	0.60
Position^c^	1.31	2.35	0.04	−3.34	5.97
IPE^d^	−4.91	3.02	−0.12	−10.90	1.07

*Note*: *R* ^2^ = 0.39, Adj *R* ^2^ = 0.37, *F* value = 14.74, Durbin–Watson statistic = 1.88, VIF Min–Max: 1.07–1.24. Analysis method = multiple regression analysis, Entry method = forced entry method, dependent variable: AITCS‐II‐J, *p < 0.001.

Abbreviations: CI, confidence interval; IPE, interprofessional professional education; JASSIC, Japanese version of Self‐assessment Scale of Interprofessional Competency; LL, lower limit; NP, nurse practitioner; SE, standard error; UL, upper limit; *β*, standardized beta value.

^a^ JASSIC range from 18 to 90, with higher scores indicating a higher ability for interprofessional collaboration.

^b^ Workplace Support Scale range from 12 to 60, with higher scores indicating a higher degree of support received from one's workplace.

^c^ 0 = Absence, 1 = Presence.

^d^ 0 = Absence, 1 = Presence.

### 3.3 Relationship between the ability of nurse practitioners to collaborate with other professionals and interprofessional collaboration in the workplace

Table 3 illustrates the relationship between the ability of NPs to collaborate with other professionals (JASSIC) and interprofessional collaboration in the workplace (AITCS‐II‐J). A multiple regression analysis was conducted with the AITCS‐II‐J as the dependent variable and the JASSIC sub‐items as the explanatory variables. The total score of the AITCS‐II‐J was related to the “Patient‐/Client‐/Family‐/Community‐Centered” (*β* = 0.31, 95% CI: 0.42–4.08) and “Facilitation Relationship” (*β* = 0.25, 95% CI: 0.19–3.43) sub‐items of the JASSIC. The adjusted *R* ^2^ value was 0.29. As a result of verifying model assumptions, the VIF values of the explanatory variables were 2.81, 3.81, 3.99, 2.27, 2.59, and 2.33 for Domains 1, 2, 3, 4, 5, and 6, respectively. There was no multicollinearity.

**TABLE 3:** Relationship between the ability of Nurse Practitioners to collaborate with other professionals and interprofessional collaboration in the workplace (*N* = 137).

JASSIC^a^ subscale	*B*	SE	*β*	95%CI	
				LL	UL
Patient‐/client‐/family‐/community‐centered	2.25	0.92	0.30*	0.42	4.07
Interprofessional communication	0.64	1.19	0.08	−1.72	3.02
Role contribution	−0.17	1.13	−0.02	−2.41	2.07
Facilitation relationship	1.81	0.81	0.25*	0.19	3.42
Reflection	0.66	1.04	0.07	−1.40	2.73
Understanding of others	−0.12	0.89	−0.01	−1.89	1.64

*Note*: *R* ^2^ = 0.32, Adj *R* ^2^ = 0.29, *F*‐value = 9.47, Durbin–Watson statistic = 1.84, VIF Min–Max: 2.26–3.99. Analysis method = Multiple regression analysis, Entry method = Forced entry method, Dependent variable: AITCS‐II‐J, *p < 0.05.

Abbreviations: CI, confidence interval; IPE, interprofessional professional education; JASSIC, Japanese version of Self‐assessment Scale of Interprofessional Competency; LL, lower limit; NP, nurse practitioner; SE, standard error; UL, upper limit; *β*, standardized beta value.

^a^ JASSIC range from 18 to 90, with higher scores indicating a higher ability for interprofessional collaboration.

### 3.4 Relationship between support from superiors in the workplace and the ability of nurse practitioners to collaborate with other professionals

Table 4 describes the relationship between support from superiors in the workplace and the ability of NPs to collaborate with other professionals (JASSIC). A multiple regression analysis was conducted with the JASSIC score as the dependent variable and the sub‐items of the Workplace Support Scale as the explanatory variables. The total JASSIC score was related to the “Informational Support” (*β* = 0.34, 95% CI: 0.10–1.51) and “Evaluative Support” (*β* = 0.30, 95% CI: 0.21–1.77) sub‐items of the Workplace Support Scale. The adjusted *R* ^2^ value was 0.15. As a result of verifying model assumptions, the VIF values of the explanatory variables were 3.29 for informational support, 2.76 for direct support, 2.08 for evaluative support, and 2.92 for emotional support, indicating no multicollinearity.

**TABLE 4:** Relationship between support from superiors in the workplace and the ability of nurse practitioners to collaborate with other professionals (*N* = 137).

Workplace Support Scale^a^ subscale	*B*	SE	*β*	95% CI	
				LL	UL
Informational support	0.80	0.35	0.33*	0.10	1.51
Direct support	−0.40	0.43	−0.12	−1.26	0.45
Evaluative support	0.99	0.39	0.29*	0.21	1.77
Emotional support	−0.42	0.65	−0.09	−1.72	0.87

*Note*: *R* ^2^ = 0.17, Adj *R* ^2^ = 0.15, *F* value = 6.60, Durbin–Watson statistic = 2.14, VIF Min–Max: 2.07–3.28. Analysis method = Multiple regression analysis, Entry method = Forced entry method, Dependent variable: JASSIC, *p < 0.05.

Abbreviations: CI, confidence interval; LL, lower limit; SE, standard error; UL, upper limit; *β*, standardized beta value.

^a^ Workplace Support Scale range from 12 to 60, with higher scores indicating a higher degree of support received from one's workplace.

## 4 DISCUSSION

This study aimed to examine and enhance the workplace interprofessional collaboration of Japanese NPs. The results suggested that interprofessional collaboration in the workplace was related to the ability of NPs to collaborate with other professionals and workplace support from superiors. To improve that ability and develop interprofessional collaboration in the workplace, this study discussed the following factors.

### 4.1 Status of interprofessional collaboration in nurse practitioners' workplaces and its related factors

In comparison with studies targeting nurses,^10^ interprofessional collaboration in the workplaces of NPs was slightly lower or equivalent in this study. Almost half of the NPs in this study were affiliated with the nursing department, but more than half were affiliated with non‐nursing departments, such as medical domains. Therefore, they were likely to experience a wider range of interprofessional collaborations. These differences between departments may affect the evaluation of interprofessional collaboration, as the way NPs work differs depending on their workplaces^32^, ^33^, ^34^; however, it is necessary to improve their ability to work with other professionals and enhance interprofessional collaboration in all workplaces.

The factors related to interprofessional collaboration included NPs' ability to collaborate with other professionals and workplace support from superiors (Table 2). In a study of Japanese ward nurses, several factors, such as IPE, years of experience, and position, were reported to promote interprofessional collaboration.^28^, ^29^ However, regarding interprofessional collaboration in the workplace, NPs' interprofessional collaboration skills and workplace support from superiors were more relevant than the abovementioned factors. The reason for this might be that more than 80% of the participating NPs had less than 8 years of experience, and the proportion of those in positions was low. Many NPs have supervisors, and it is possible that the inexperienced ones receive workplace support from their supervisors while improving their ability to collaborate with other professionals.

### 4.2 Relationship between the ability to collaborate with other professionals and interprofessional collaboration in the workplace

Among the NPs' interprofessional collaboration skills in this study, “Patient‐/Client‐/Family‐/Community‐Centered” and “Facilitation Relationship” were related to interprofessional collaboration in the workplace (Table 3). “Patient‐/Client‐/Family‐/Community‐Centered” describes the ability to prioritize patient concerns and issues, help families and communities, and support the setting of common team goals rather than individual specialist ones.^16^ Studies have shown that NPs were directly involved with patients and families and practiced patient‐ and family‐centered healthcare.^23^, ^35^ They also facilitate high‐quality and timely communication by capturing a wide range of patient information from the medical and nursing perspectives^23^, ^24^ while acting as a bridge between multiple professionals.^17^, ^35^ Considering this in practice may enhance NPs' ability to collaborate with other professionals regarding the “Patient‐/Client‐/Family‐/Community‐Centered” aspect and improve interprofessional collaboration in the workplace.

“Facilitation Relationship” describes the ability to grow with multiple professionals, build relationships as equals, and avoid relationship conflicts.^7^ One study reported that NPs maintain good cooperation with various professionals.^17^, ^23^ We speculated that this kind of practice will also improve interprofessional collaboration in the workplace.

However, in this study, the NPs' interprofessional collaboration skills other than “Patient‐/Client‐/Family‐/Community‐Centered” and “Facilitation Relationship” did not show a significant relationship with interprofessional collaboration in the workplace. “Interprofessional Communication,” “Role Contribution,” “Reflection,” and “Understanding of Others” are abilities that allow professionals to convey their opinions and fulfill their roles.^7^ This ability includes qualities that are enhanced by gaining sufficient and extended experience in an organization. It is possible that the NPs in this study had not yet had as much experience. By improving these skills while gaining support from their superiors, NPs' interprofessional collaboration in the workplace can improve.

### 4.3 Types of workplace support related to nurse practitioners' ability to collaborate with multiple professionals

To enhance NPs' ability to collaborate with other professionals, this study analyzed workplace support (Table 4). NPs' ability to collaborate with other professionals was related to workplace, informational, and evaluative support, consistent with previous studies showing a relationship with organizational (workplace) support.^36^ However, this kind of support has not been studied extensively. Nevertheless, these results provide new insights into how NPs can improve their ability to collaborate with other professionals.

Informational Support involves providing the knowledge and information necessary to solve work‐related problems.^37^ We assumed that NPs' ability to collaborate with other professionals would improve if they obtained the knowledge and information necessary to solve work‐related problems from their superiors. Additionally, the role of NPs is not always clearly defined in the workplace.^24^ Studies have shown that role clarity is important for smooth interprofessional collaboration^38^; therefore, supervisors must provide NPs with information and support, such as role clarity.^39^

Evaluative Support refers to the evaluation and approval of ability and effort.^37^ This study assumed that the ability of NPs to collaborate with other professionals would improve if they received an evaluation from their superiors regarding their abilities and efforts in this area. Approval from medical staff leads to the strengthening of smooth relationships,^40^ whereas that from superiors improves professional abilities and growth.^39^ Therefore, NPs can improve their abilities by collaborating with other professionals and gaining approval from their superiors.

## 5 LIMITATIONS

This study evaluated NPs' interprofessional collaboration through self‐assessment but did not evaluate professionals besides NPs. The results of the combinations with other professionals should be studied in the future. It is also necessary to improve our understanding of how interprofessional collaboration can be enhanced. Moreover, the response rate was low (18%), indicating possible respondent and sampling biases. Thus, the results' generalizability must be interpreted with caution.

## 6 CONCLUSIONS

An analysis of multidisciplinary collaboration among NPs in Japan revealed that interprofessional collaboration within teams is associated with NPs' interprofessional collaboration skills and workplace support. Additionally, regarding workplace support, informational and evaluative support were found to be related, and suggestions were provided on how supervisors can effectively engage with NPs to bring out their abilities. These findings highlight specific measures to promote interprofessional collaboration among NPs in Japan.

## 7 AUTHOR CONTRIBUTIONS

**Naoko Tokunaga:** Conceptualization; Data curation; Formal analysis; Investigation; Methodology; Project administration; Visualization; Writing – original draft; Writing – review and editing. **Akihiro Araki:** Supervision. **Hiromi Fukuda:** Supervision, Writing – review and editing.

## FUNDING INFORMATION

Allocated by the university.

## CONFLICT OF INTEREST STATEMENT

Authors declare no conflict of interests for this article.

## ETHICS STATEMENT

Ethics approval statement: The study was approved by the Ethical Committee of the Oita University of Nursing and Health Sciences Research (approval number 23‐54).

Patient consent statement: The consent form explained in writing that participants' responses to the questionnaire would be considered consent.

Clinical trial registration: None.

## Data Availability

As a result of the nature of this research, participants of this study did not agree for their data to be shared publicly, so supporting data is not available.
